# Mitogen-activated protein kinase p38 modulates pacemaker ion channels differentiation in P19-derived pluripotent cells

**DOI:** 10.1186/s12576-020-00766-x

**Published:** 2020-09-07

**Authors:** Mingqi Zheng, Lin Kang, Tomoko Uchino, Gang Liu, Yan Wang, Katsushige Ono

**Affiliations:** 1 Department of Pathophysiology, Oita University School of Medicine, Oita, Japan; 2 Department of Cardiovascular Medicine, Hebei Medical University, Shijiazhuang, Hebei China; 3 Department of Anatomy, Hebei Medical University, Shijiazhuang, Hebei China; 4 Department of Anesthesiology, Oita University School of Medicine, Oita, Japan

**Keywords:** Csx/Nkx2.5, GATA4, MEF2C, p38MAP kinase, Cardiogenesis, P19CL6

## Abstract

Signal regulators during early cardiogenetic differentiation for the cellular automaticity are largely unknown. Our investigations were designed to clarify the role of transcription factors and their modulators in P19-derived cardiomyocytes to the expression of cardiac pacemaker ion channels. Transcription factors Csx/Nkx2.5 and GATA4 but not MEF2C were markedly inhibited by p38 MAP kinase inhibition in a distinct manner; expression but not phosphorylation of GATA4 was reduced by inhibition of p38 MAP kinase actions. In the presence of an ERK1/2,5 inhibitor PD98059 or a JNK MAP kinase inhibitor SP600125, P19 cells successfully differentiated into cardiomyocytes displaying spontaneous beatings with expression of three types of pacemaker ion channels. We demonstrate that acquisition of cellular automaticity and the expression of pacemaker ion channels are regulated by the transcription factors, Csx/Nkx2.5 and GATA4, through intracellular signals including p38 MAP kinase in the process of P19-derived pluripotent cells differentiation into cardiomyocytes.

## Background

The generation of pacemaker ionic channels is regarded as one of the most important factors responsible for the spontaneous beating of myocytes. To date, many studies have demonstrated several ionic mechanisms for the generation of slow diastolic depolarization in action potentials at the sino-atrial node in the heart [1, 2], including the L-type Ca^2+^ channel current (*I*_Ca.L_), the T-type Ca^2+^ channel current (*I*_Ca.T_) [3], the hyperpolarization-activated inward current (*I*_f_) [4], the rapidly activating delayed rectifier K^+^ current (*I*_Kr_) [5], and the sustained inward current (*I*_st_) [6].

In mammalian embryonal differentiation, morphogenesis of the cardiovascular system is initiated in 20 gestational days in humans and 7.5 gestational days in mice, followed by the formation of a simple tubular heart and generation of spontaneous beating [7]. The generation of specific contractile proteins and ion channel proteins in earlier cardiac differentiation is triggered by cardiac-specific gene expression. It has recently been shown that the expression of cardiac genes is regulated by several specific transcription factors during embryonal heart differentiation [8, 9]. Csx/Nkx2.5, GATA4, and MEF2C are considered to be cardiac-specific transcription factors that play the critical roles in the early development of the heart, and serve as useful molecular markers to identify cardiac inductive signals from other tissues or germ layers [10].

The homeobox gene Csx/Nkx2.5, a vertebrate homologue of *Drosophila tinman*, is one of the earliest known markers of mesoderm that give rise to myocardium, and its expression persists throughout the myocardium in the fully formed heart [7, 8]. GATA4 serves as a cardiac-specific member of the GATA family of zinc finger transcription factors which is detected very early in the cardiogenetic area and persists later in the developing heart [11]. MEF2C belongs to the MEF2 family of MADS-box, and is expressed throughout the heart during mouse embryogenesis which controls cardiac morphogenesis and myogenesis [8, 12]. In the stimulation of cellular proliferation factors, including BMP, ET-1, and CT-1, the differentiation of cardiomyocytes is generated via the activation of intranuclear transcription factors such as Csx/Nkx2.5, GATA4, and MEF2C [7, 10]. However, in the early stage of embryonal development, signal transduction systems from the cellular membrane receptor signals into intranuclear transcription factors, subsequently activate the cardiac-specific proteins including ion channel expression, are poorly understood. On the other hand, the MAP kinase pathways, ERK1/2, ERK5, JNK, and p38 superfamilies, are major signaling systems by which the cells transfer extracellular signals into intracellular responses [13]. It is known that MAP kinase performs the key roles in the differentiation of cardiomyocytes and in morphogenesis [14].

P19CL6 cells, clonal derivatives of mouse pluripotent P19 embryonal carcinoma cells, efficiently differentiate into spontaneously beating cardiomyocytes under adherent conditions when treated with 1% dimethyl sulfoxide (Me_2_SO) [10, 15]. It is widely accepted that P19 cells are excellent models for studying the development of cardiogenesis and cardiac gene expression in more than 100 studies [10, 15–18]. In this context, we focused on the molecular mechanisms of the expression of cardiac ionic channels, in order to assess the possible interaction of MAP kinase and specific cardiac transcription factors in P19-derived cardiomyocytes. We show that the expression of pacemaker ion channels are regulated by the transcription factors, Csx/Nkx2.5 and GATA4, through intracellular signals including p38 MAP kinase in the process of P19 cells differentiation into cardiomyocytes with automaticity.

## Materials and methods

### Cell culture and differentiation

P19CL6 cells were cultured with α-MEM containing 1% dimethyl sulfoxide (Me_2_SO) to induce differentiation into spontaneously beating cardiomyocytes as described previously [10, 15], with or without one of the following MAPK inhibitors: PD98059 (PD), an inhibitor of ERK1/2,5; SP600125 (SP), an inhibitor of JNK; SB203580 (SB), a specific inhibitor of the p38-pathway. For long-term inhibition of MAP kinase signals, 10 μM concentration of each inhibitor was applied [17, 18]. Days of differentiation were numbered consecutively after the first day of 1% Me_2_SO treatment, considered as day 0.

### Single cardiomyocyte preparation

Single P19-derived cardiomyocytes were isolated from differentiated beating cardiomyocytes in a 60-mm tissue culture dish on days 14 to 17 using a modified procedure described previously [10, 15].

### Electrophysiological recordings

Spontaneously beating single cardiomyocytes were chosen for electrophysiological recordings by using of whole-cell current clamp and voltage clamp experiments [4, 19, 20]. All electrophysiological measurements were performed at 36 °C. Data were acquired at a sampling rate of 10 kHz, filtered at 2 kHz, and analyzed off-line by using the Pulsefit (HEKA) and Sigma9.0 software (SPSS Inc. Chicago, IL, USA).

### Solutions and chemicals

For measuring membrane potentials, the bath solution (normal Tyrode solution) contained (in mM) NaCl 140, KCl 5.4, CaCl_2_ 1.8, MgCl_2_ 1, HEPES 10, and glucose 10, with the pH adjusted to 7.4 with NaOH. The pipette solution contained (in mM) KCl 140, MgCl_2_ 2, Mg-ATP 5, creatine phosphate (disodium salt) 5, EGTA 0.05, and HEPES 10, with the pH adjusted to 7.2 with KOH. For measuring *I*_Ca_, the bath solution contained the normal Tyrode solution supplemented with 30 μM tetrodotoxin to eliminate Na^+^ current. The pipette solution contained (in mM) CsCl 130, MgCl_2_ 2, Mg-ATP 2, Na_2_-GTP 2, EGTA 10, and HEPES 5, with the pH adjusted to 7.2 with CsOH. For measuring *I*_f_, the bath solution contained the normal Tyrode solution supplemented with (in mM) BaCl_2_ 1, CdCl_2_ 0.4, tetrodotoxin 0.03, 4,4′-diisothiocyanostilbene-2,2′-disulfonic acid (DIDS) 0.1, and 4-aminopyridine (4-AP) 1, to eliminate inward rectifier K^+^ current, Ca^2+^ current (T- and L-type), Na^+^ current, and transient outward K^+^ current (*I*_to_), respectively. The pipette solution contained (in mM) KCl 140, MgCl_2_ 1, EGTA 10, HEPES 5, and Mg-ATP 5, with the pH adjusted to 7.2 with KOH. PD98059, SP600125, and SB203580 were purchased from Calbiochem (La Jolla, CA, USA). All other chemicals were purchased from Wako Pure Chemical Industries (Osaka, Japan).

### Quantitative real-time RT-PCR

Total RNA was extracted from P19-derived cardiomyocytes using Isogen (Nippongene, Tokyo, Japan). The cDNA was synthesized from 1 μg of total RNA using Transcriptor First Strand cDNA Synthesis Kit (Roche Molecular System Inc., Alameda, CA, USA). The real-time PCR was performed on Light Cycler (Roche) using the FastStart DNA Master SYBR Green I (Roche) as a detection reagent. For Csx/Nkx2.5, primer F sequenced 5′-CAG TGG AGC TGG ACA AAG CC-3′, and primer R sequenced 5′-TAG CGA CGG TTC TGG AAC CA-3′ were used. For GATA4, primer F sequenced 5′-GCC TGT ATG TAA TGC CTG CG-3′, and primer R sequenced 5′-CCG AGC AGG AAT TTG AAG AGG -3′ were used. For MEF2C, primer F sequenced 5′-GTA TGT CTC CTG GTG TAA CA-3′, and primer R sequenced 5′-GGA TAT CCT CCC ATT CCT TG-3′ were used. As an internal control in each reaction, mouse GAPDH primer F of 5′-CCA AGG TCA TCC ATG ACA AC-3′, and primer R of 5′-TTA CTC CTT GGA GGC CAT GT-3′ were used. The expression of each target mRNA relative to GAPDH under experimental and control conditions was calculated based on the threshold cycle (CT) as r = 2^−Δ(ΔCT)^, where ΔCT = CT _target_ − CT _GAPDH_ and Δ(ΔCT) = ΔCT _experimental_ − ΔCT _control_. Gel electrophoresis was performed to confirm the correct size of amplification and the absence of unspecific bands.

### Western blot analysis

P19-derived cardiomyocytes were lysed in cold cell lysis buffer. The lysates were clarified by centrifugation at 12 000*g* for 15 min at 4 °C. The supernatants were used for phospho-p38 and total p38 Western blot analysis.

### Analysis of data

All data are presented as mean ± S.E. One-way ANOVA followed by a Bonferroni post hoc test was used for multiple comparisons. Differences were considered significant when *p* values were less than 0.05.

## Results

### Action potentials in differentiating P19-derived cells

**Fig. 1:**
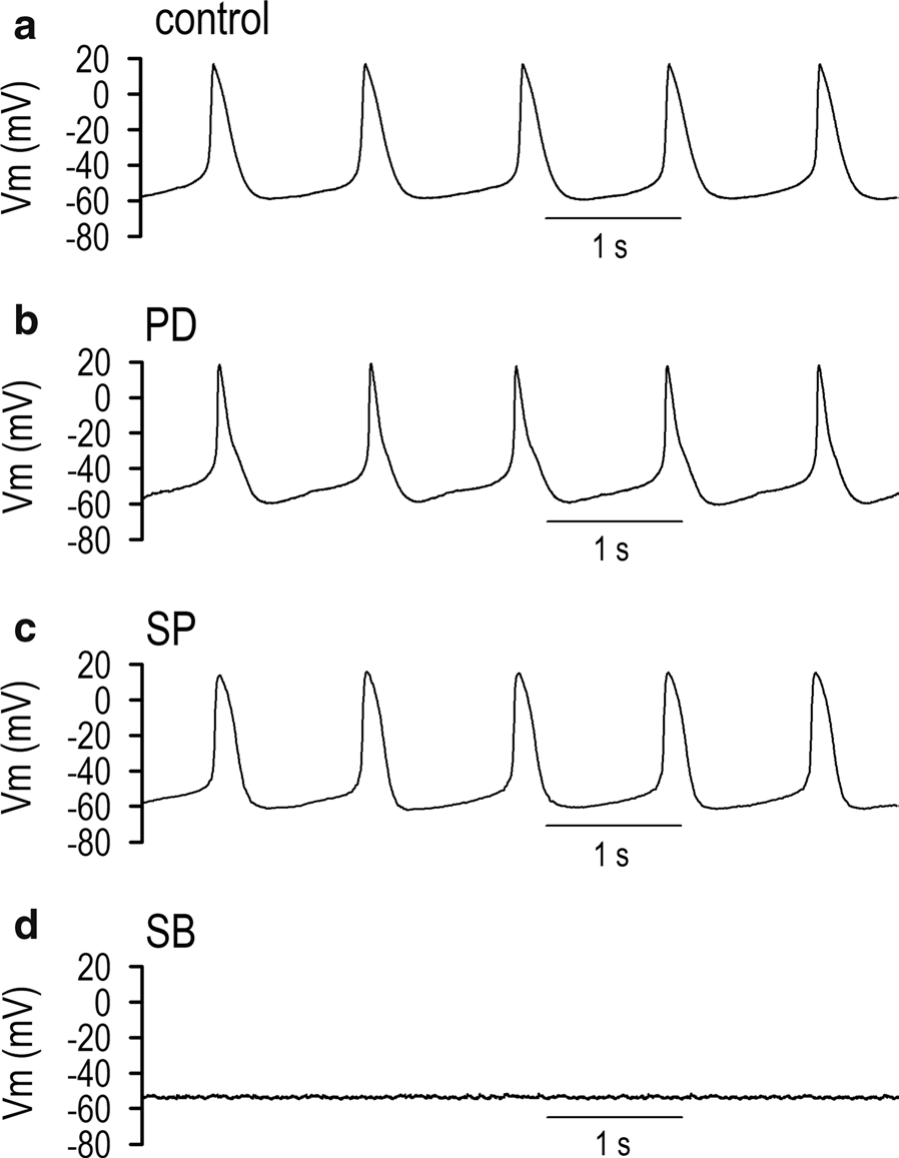
Action potentials in differentiating P19 cells. **a** Representative action potentials (APs) from a single isolated beating cardiomyocytes derived from P19 cells on differentiation day 14 with vehicle (control). Note that the APs exhibit distinct spontaneous diastolic depolarization with the maximum diastolic potential (MDP) of − 58.2 mV. **b–d** Representative action potentials (membrane potentials) in P19-derived cardiomyocytes on differentiation day 14 in the presence of 10 μM PD98059 (**b**), 10 μM SP600125 (**c**), and 10 μM SB203580 (**d**) recorded by the same protocol described in **a**

Spontaneous beating activity is thought of as the unique character of the cardiomyocyte, distinct from other cells. We investigated mechanisms for the spontaneous beating activity of P19-derived cardiomyocytes by means of electrical excitability or action potentials. P19-derived cardiomyocytes displayed spontaneous beating, which was initialized on differentiated day 10 with very slow frequency. The beating rates reached 89.2 ± 17.4 bpm (*n* = 15) on days 14 to 17 (Table 1). Action potentials (APs) measured by current-clamp mode indicated a maximum diastolic potential (MDP) of − 58.6 ± 2.5 mV (*n* = 15) on days 14 to 17 (Fig. 1a). On the other hand, in the absence of Me_2_SO, P19 cells did not differentiate into cardiomyocytes, thus demonstrating no spontaneous beating activity (Table 1). The membrane potentials (MP) were − 52.8 ± 8.5 mV (*n* = 11) on differentiated day 0 (data not shown) and − 51.9 ± 6.7 mV (*n* = 14) on days 14 to 17.

**Table 1:** Parameters of action potentials on day 14–17

	α-MEM (*n* = 14)	1% Me_2_SO (*n* = 15)	1% Me_2_SO + PD (*n* = 12)	1% Me_2_SO + SP (*n* = 11)	1% Me_2_SO + SB (*n* = 11)
BR (bpm)	0	89.2 ± 17.4	82.7 ± 11.8	95.7 ± 12.3	–
MDP (mV)	− 51.9 ± 6.7	− 58.6 ± 2.5	− 57.9 ± 1.7	− 58.1 ± 3.1	− 49.0 ± 3.3*
APD_50_ (ms)	–	138 ± 7	105 ± 9*	149 ± 11	–
APD_75_ (ms)	–	278 ± 14	299 ± 19	247 ± 17	–

PD: PD98059, 10 μM; SP: SP600125, 10 μM; SB: SB203580, 10 μM

APD_50_, the duration of 50% repolarization of action potentials

APD_75_, the duration of 75% repolarization of action potentials

*BR* beating rates, *MDP* maximum diastolic potentials

Mean ± S.E. *p < 0.05 vs. control (1% Me_2_SO)

To clarify the intracellular signals in the cardiogenetic process, MAP kinase-dependent signal transduction systems were explored in accordance with the acquisition of cellular excitability. We first examined the possible contribution of ERK1/2- and ERK5-dependent cascades to the development of action potential configurations. PD98059 (PD) has been widely used as a selective inhibitor of ERK1/2,5, which belong to MAP kinase kinase superfamilies. We recorded the action potentials of P19-derived cells on differentiation days 14 to 17, the cells of which were treated with PD throughout the cardiogenetic process from day 0. Similar to the control condition (1% Me_2_SO without MAPK kinase inhibitor), P19-derived cardiomyocytes displayed  spontaneous beating of 83 ± 12 bpm, MDP of − 57.9 ± 1.7 mV (Fig. 1b), APD_50_ of 105 ± 9 ms, and APD_75_ of 299 ± 19 ms in the presence of PD (Table 1). To study the possible interaction of the JNK pathway, another branch of MAP kinase cascade, we used SP600125 (SP) in order to selectively abolish the JNK MAP kinase-dependent signal cascade. We found that action potentials were distinctly formed (Fig. 1c), and their parameters of APs were similar to those observed under control conditions (Table 1). In the presence of SB203580 (SB), an inhibitor of p38, P19 cells were found to be quiescent without spontaneous beating during the observation period of 30 days. The measured MDP (− 49.0 ± 3.3 mV, *n = *11) was statistically identical to that of undifferentiated P19 cells (− 51.9 ± 6.7 mV, *n* = 14) on days 14 to 17 (Fig. 1d and Table 1). To identify the detailed electrophysiological development of P19-derived cells in the presence of PD, SP, or SB, we initially focused on parameters of membrane potentials: APD_50_, APD_75_, and MDP (Table 1). The APD_50_ in Me_2_SO + PD-treated cells were significantly shorter, and maximum diastolic potentials in Me_2_SO + SB treated cells were significantly depolarized in comparison with that in Me_2_SO treated cells. Other parameters in PD and SP groups were all statistically identical to those in Me_2_SO-treated cells.

### Electrophysiological characters of pacemaker channel currents in differentiated P19 cells

**Fig. 2:**
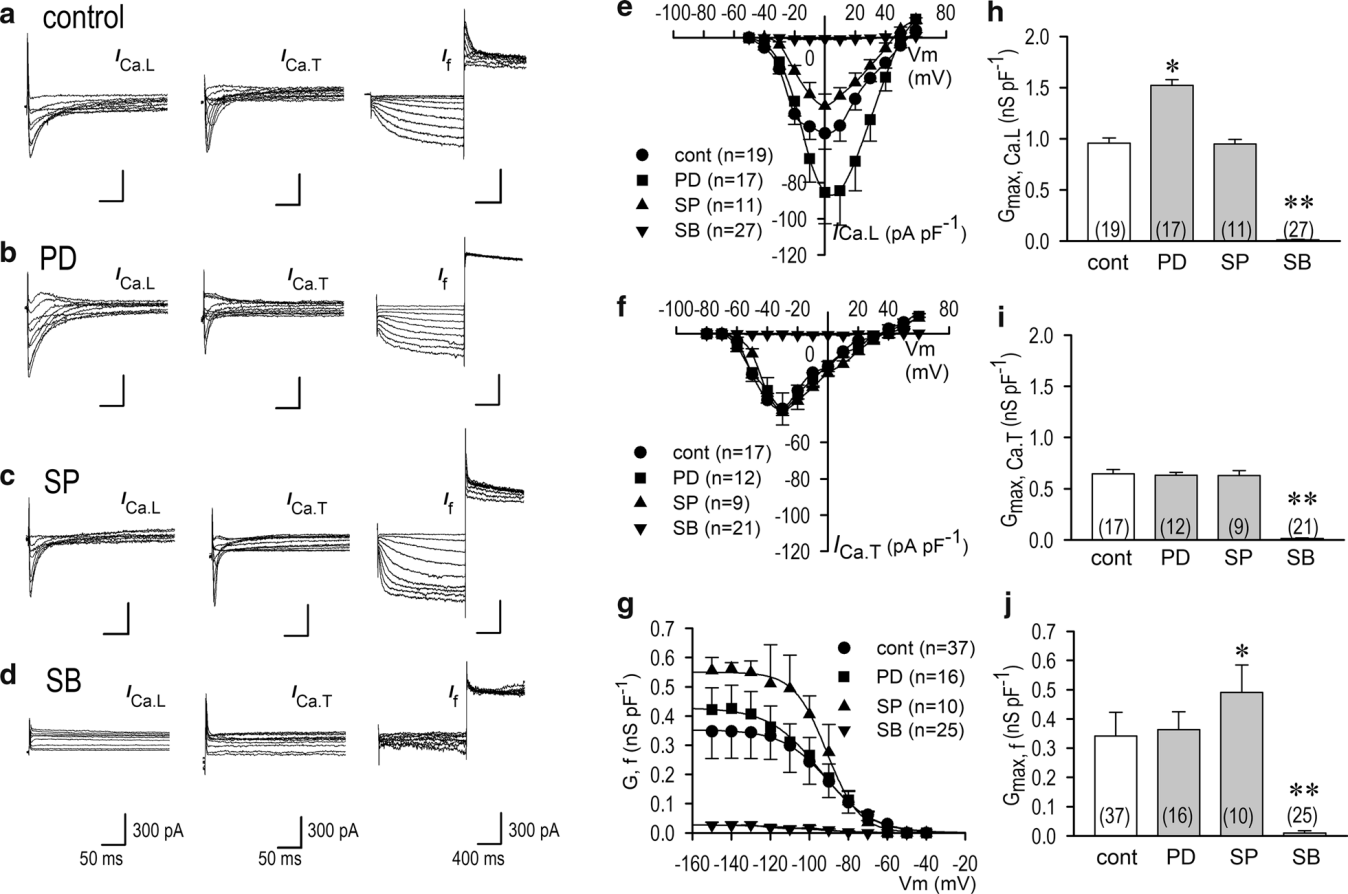
Current traces of *I*_Ca.L_, *I*_Ca.T_ and hyperpolarization-activated inward current (*I*_f_) in P19-derived cardiomyocytes and Effects of MAPK inhibitors. **a** Representative current traces of *I*_Ca.L_, *I*_Ca.T_ and *I*_f_ from a single isolated beating cardiomyocytes derived from P19 cells on differentiation day 14 with vehicle (control). *I*_Ca.T_ was recorded by subtracting the current traces obtained by *V*_HP_ of − 50 mV from the current traces obtained by *V*_HP_ of − 100 mV. *I*_f_ was obtained from *V*_HP_ of − 40 mV to various test potentials ranging from − 50 to − 140 mV in 10 mV increment. **b–d** Representative current traces of *I*_Ca.L_, *I*_Ca.T_ and *I*_f_ in P19-derived cardiomyocytes on differentiation day 14 in the presence of 10 μM PD98059 (**b**), 10 μM SP600125 (**c**), and 10 μM SB203580 (**d**). **e**–**g** The graphic representation of the current (*I*)–voltage (*V*) relationships of *I*_Ca.L_ (**a**), *I*_Ca.T_ (**b**), and conductance (*G*)–voltage (*V*) relationship of *I*_f_ (**g**). **h** The maximum chord conductance of *I*_Ca.L_ (*G*_max_, _Ca.L_) obtained at the potential range from + 20 mV to + 40 mV. **i** The maximum chord conductance of *I*_Ca.T_ (*G*_max_, _Ca.T_) obtained at the potential range from − 10 mV to + 10 mV. **j** The maximum chord conductance of *I*_f_ (*G*_max_, _f_) obtained at the potential range from − 120 to − 150 mV. Current amplitude of *I*_Ca.L_ and *I*_Ca.T_ were assessed by means of time-dependent components of each trace. *p < 0.05 vs. control, **p < 0.01 vs. control. Filled circle, control (*n* = 17–37); filled square, 10 μM PD98059 (*n* = 12–17); filled upward triangle, 10 μM SP600125 (*n* = 9–11); filled downward triangle, 10 μM SB203580 (*n* = 21–27)

We investigated the development of membrane currents focusing on pacemaker channel currents such as L-type Ca^2+^ current (*I*_Ca.L_), T-type Ca^2+^ current (*I*_Ca.T_), and hyperpolarization-activated inward current (*I*_f_). Expression of three types of pacemaker currents was identified in the P19-derived cardiomyocytes on differentiation days 14 to 17 (Fig. 2a). However, under the differentiating condition from day 0 to day 7, these three types of ion channels were not detected (data not shown), which was consistent with quiescence of the cells on those days. In the presence of PD and SP, *I*_Ca.L_, *I*_Ca.T_ and *I*_f_ were all distinctly formed in function (Fig. 2b, c), which were nearly identical to those under control conditions (Fig. 2a). In the presence of SB, these ionic currents were not expressed in function (Fig. 2d). Subsequently, we investigated *I*_Ca.L_ in terms of current density and voltage dependency. All of the *I*_Ca.L_ values recorded, except those in the presence of SB, demonstrated a similar current (*I*)–voltage (*V*) relationship (Fig. 2e). The maximum peak currents were obtained at the test potential of 0 mV. The maximum conductance for the L-type Ca^2+^ channel (*G*_max, Ca.L_) were 0.96 ± 0.05 nS pF^−1^ (*n* = 19) under control conditions, 1.52 ± 0.06 nS pF^−1^ (*n* = 17) in the presence of PD, and 0.95 ± 0.06 nS pF^−1^ (*n* = 11) in the presence of SP (Fig. 2h), whereas *I*_Ca.L_ was completely abolished by SB (Fig. 2e, h, Table 2). In the presence of PD, *I*_Ca.L_ and *G*_max, Ca.L_ were significantly larger than those in control by unknown reasons.

**Table 2:** Electrophysiological characteristics of three pacemaker ionic currents and cellular membrane capacitance on the differentiation day 14–17

	α-MEM	1% Me_2_SO	1% Me_2_SO + PD	1% Me_2_SO + SP	1% Me_2_SO + SB
*I*_Ca.L_	0	− 52.1 ± 8.8	− 85.4 ± 10.6	− 1.6 ± 6.4	0
(pA pF^−1^)	(*n* = 11)	(*n* = 19)	(*n* = 17)*	(*n* = 11)*	(*n* = 27)*
*I*_Ca.T_	0	− 41.4 ± 17.6	− 43.1 ± 10.9	− 42.1 ± 9.5	0
(pA pF^−1^)	(*n* = 10)	(*n* = 17)	(*n* = 12)	(*n* = 9)	(*n* = 21)*
*I*_f_	0	− 26.9 ± 4.7	− 28.5 ± 3.9	− 37.4 ± 6.3	− 0.1 ± 0.2
(pA pF^−1^)	(*n* = 8)	(*n* = 37)	(*n* = 16)	(*n* = 10)*	(*n* = 25) *
*C*_m_	7.0 ± 1.9	20.1 ± 5.2	21.4 ± 6.8	19.2 ± 7.2	11.9 ± 3.3
(pF)	(*n* = 11)	(*n* = 62)	(*n* = 41)	(*n* = 41)	(*n* = 67) *

PD: PD98059, 10 μM; SP: SP600125, 10 μM; SB: SB203580, 10 μM

*C*_m_, cellular membrane capacitance

Mean ± S.E. *p < 0.05 vs. control (1% Me_2_SO)

The I–*V* relationship of *I*_Ca.T_ indicated that the maximum conductance for the T-type Ca^2+^ channel (*G*_max, Ca.T_) obtained at − 30 mV was 0.64 ± 0.04 nS pF^−1^ (*n* = 17) under control conditions, and was not affected by the presence of PD or SP, that is to say, *G*_max, Ca.T_ and the maximum peak current of *I*_Ca.T_ under the effect of PD or SP were highly similar to those under control conditions, whereas *I*_Ca.T_ was completely abolished by SB (Fig. 2f, i, Table 2).

*I*_f_ densities at − 120 mV were − 26.9 ± 4.7 pA pF^−1^ (*n* = 37) under control conditions, − 28.5 ± 3.9 pA pF^−1^ (*n* = 16) in the presence of PD, and − 37.4 ± 6.3 pA pF^−1^ (*n* = 10) in the presence of SP, indicating no statistically different significance between control group and PD group. Conductance curves for *I*_f_ indicate that half-activated potentials (*V*_1/2_) were − 94.5 ± 3.4 mV under control conditions, − 90.1 ± 5.6 mV in the presence of PD, and − 89.4 ± 5.3 mV in the presence of SP (Fig. 2g). *I*_f_ and G_max,f_ were significantly larger in SP group (Fig. 2g, j). However, in the presence of SB, *I*_f_ displayed substantially no inward currents like to those of *I*_Ca.L_ and *I*_Ca.T_. Interestingly, under the effect of SB, P19 cells were retarded in development in terms of membrane capacitance (Table 2), indicating a significant role of p38 in cell differentiation and growth.

### Transcription factor changes in differentiated P19 cells

**Fig. 3:**
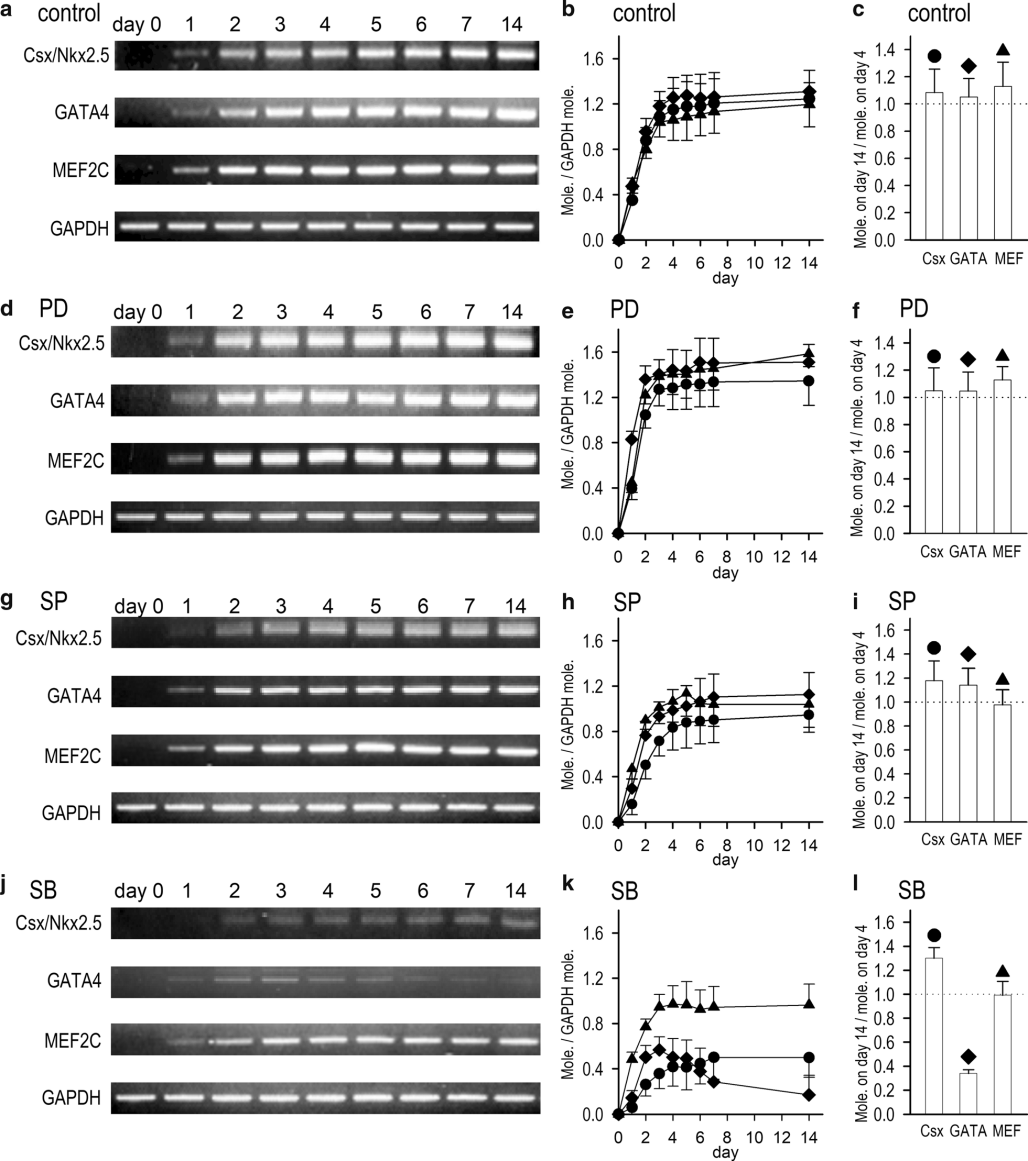
Cardiac-specific transcription factors expression in P19-derived cardiomyocytes. **a** RT-PCR products of Csx/Nkx2.5, GATA4, and MEF2C prepared from P19-derived cardiomyocytes on differentiation day 0 to 7 and day 14 with vehicle (control). **b** Time course of mRNA expression of Csx/Nkx2.5 (filled circle), GATA4 (filled diamond) and MEF2C (filled upward triangle) normalized to GAPDH mRNA in the differentiation period. **c** Fractional mRNA expression of Csx/Nkx2.5, GATA4 and MEF2C on day 14, normalized to those on day 4. RT-PCR products (**d**, **g**, **j**) and their time course (**e**, **h**, **k**) of Csx/Nkx2.5, GATA4, and MEF2C prepared from P19-derived cardiomyocytes on differentiation day 0 to 7 and day 14 in the presence of 10 μM PD98059, 10 μM SP600125, and 10 μM SB203580. **f**, **i**, **l** Fractional mRNA expression of Csx/Nkx2.5, GATA4 and MEF2C on day 14, normalized to those on day 4 in the presence of PD98059 (**f**), SP600125 (**i**), and SB203580 (**l**)

In order to identify the signals to initiate ion channel expression in terms of transcription factor activation, we performed RT-PCR analyses for the detection of the mRNA encoding cardiac-specific transcription factors Csx/Nkx2.5, GATA4, and MEF2C which may be responsible for the expression of ion channels. Three cardiac-specific transcription factors appeared as early as day 1 in the differentiation stage, and their intensive expression were successively detected in the presence of Me_2_SO (Fig. 3a). Importantly, expression of Csx/Nkx2.5, GATA4 and MEF2C were quantitatively identical in amount and nearly stable from day 4 to day 14 (Fig. *3*b, c).

We also found that under the effect of an ERK1/2,5-inhibitor PD, the expression of the three specific transcription factors was appreciably increased, with the same time course (Fig. 3d–f) as that under control conditions (Fig. *3*a–c). Simultaneously, expression of cardiac-specific transcription factors under the effect of a JNK MAP kinase inhibitor SP was almost identical to those under control conditions in terms of time course and total RNA molecules (Fig. 3g–i). Therefore, JNK MAPK kinase was identified as not modifying the cardiogenetic process in terms of cardiac pacemaker ion channels expression. Csx/Nkx2.5 mRNA was slowly and suppressively expressed by a p38 inhibitor SB. Importantly, GATA4 mRNA was declined on differentiation day 4 or later by SB (Fig. 3l). On the other hand, MEF2C mRNA expression was unaffected by a p38 inhibitor SB. These results indicate that the expression of cardiac pacemaker ion channels and the beating property of myocytes are distinctly controlled by specific transcription factors, Csx/Nkx2.5 and GATA4, activated by the p38 MAP kinase pathway.

**Fig. 4:**
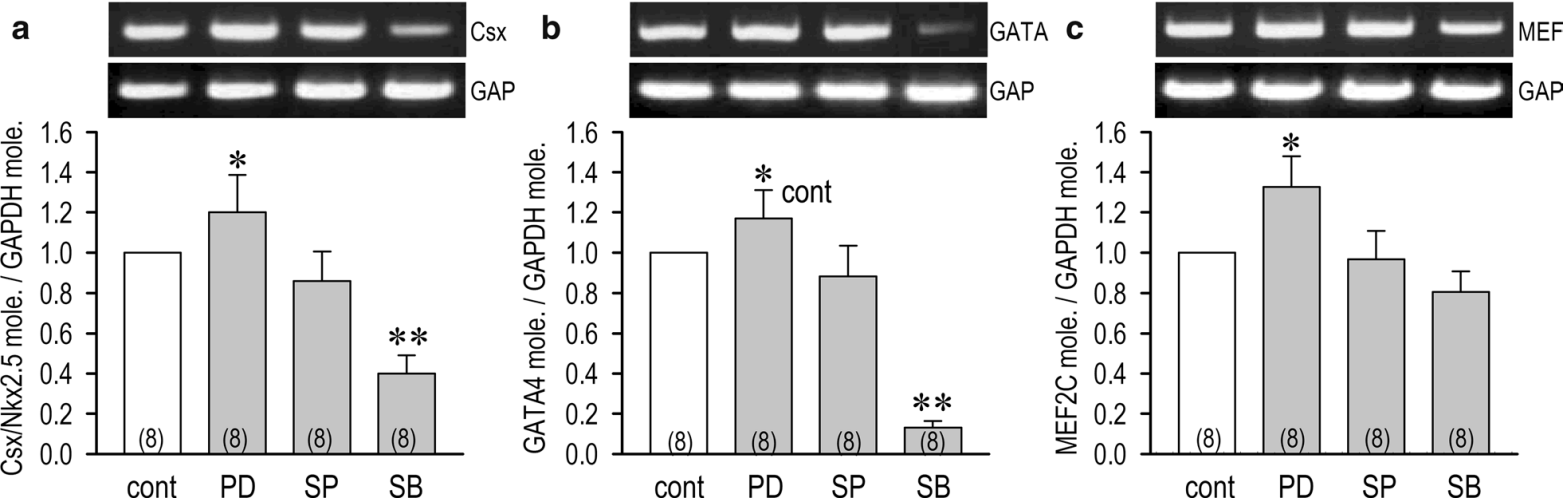
Effects of MAPK inhibition on cardiac-specific transcription factors expression. Quantitative analyses in RT-PCR for three transcription factors Csx/Nkx2.5 (**a**), GATA4 (**b**), and MEF2C (**c**), were performed on the differentiation day 14 with or without MAP kinase inhibitors (control): PD98059 (10 μM), SP600125 (10 μM) and SB203580 (10 μM). Each expression level was normalized to the ratio in control cells on the differentiation day 14. Representative PCR products are shown in inset (upper) with the reference gene GAPDH (below). Each mRNA product was normalized to that of GAPDH. Data were calculated and normalized to those in the control condition (without MAP kinase inhibitor). *p < 0.05 vs. control, **p < 0.01 vs. control

Expression of transcription factors were quantitatively compared in the presence of MAP kinase inhibitors on the differentiation day 14 (Fig. 4). Expression of Csx/Nkx2.5 was halved and expression of GATA4 was highly inhibited in the presence of SB (Fig. 4a, b). On the other hand, the expression levels of Csx/Nkx2.5, GATA4 and MEF2C were all significantly increased by PD (Fig. 4a–c). Taken together, p38 MAP kinase-dependent signal transduction and classical MAP kinase 1/2,5 signal transduction contrarily modulate transcription factors Csx/Nkx2.5 and GATA4 in the process of cardiogenetic differentiation.

### P38 protein changes in differentiated P19 cells

**Fig. 5:**
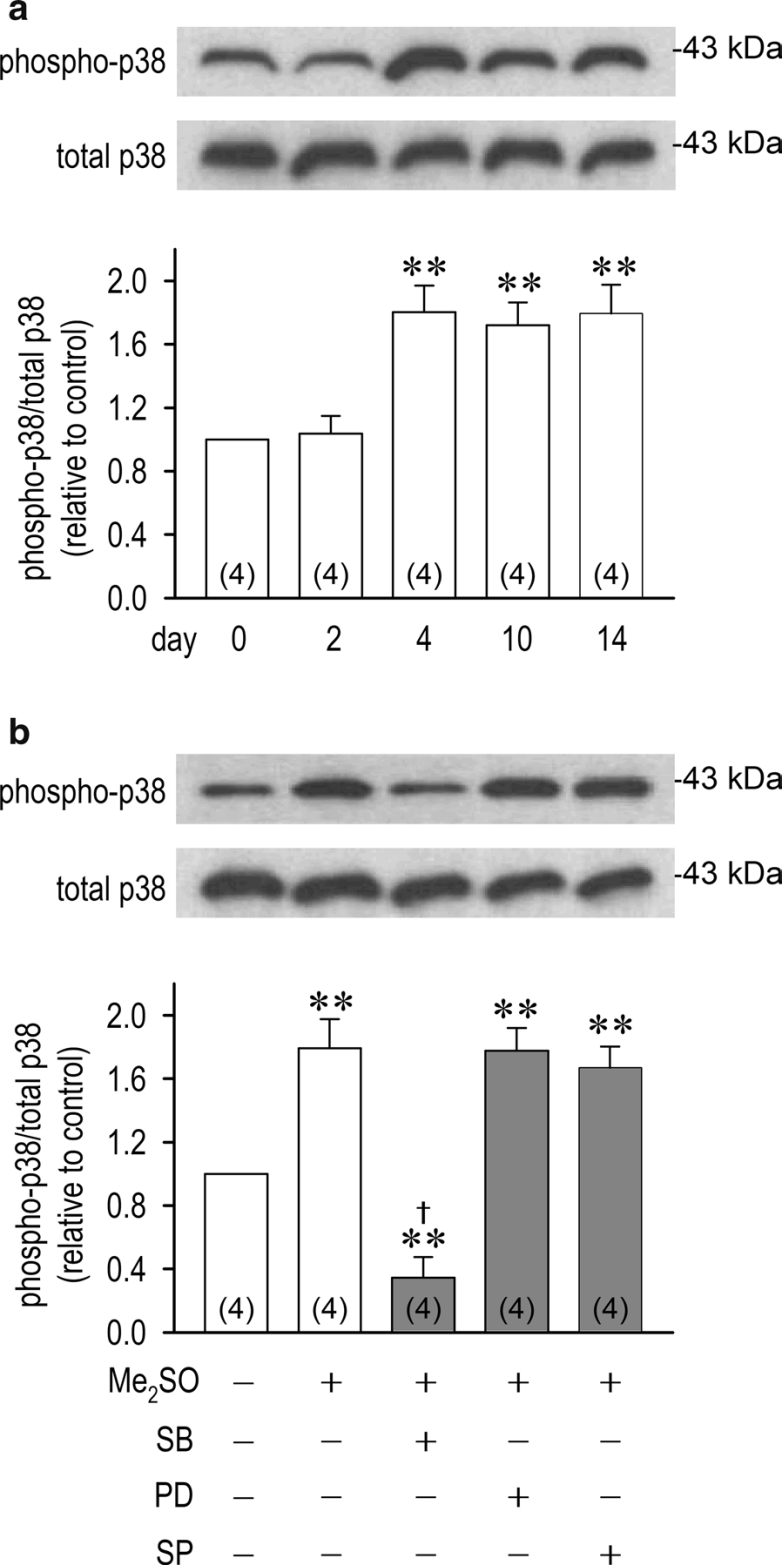
Effects of MAPK inhibition on p38 and phospho-p38 proteins expression. **a** Time-dependent changes of p38 and phospho-p38 proteins extracted from P19-derived cardiomyocytes assessed by Western blot analysis. **b** p38 and phospho-p38 proteins expression on the differentiation day 14 with or without MAP kinase inhibitors: PD98059 (10 μM), SP600125 (10 μM) and SB203580 (10 μM). The results in bar graphs are expressed as the ratio of phosphorylated proteins to total proteins. **p < 0.01 vs. day 0 or Me_2_SO (−). †*p* < 0.01 vs. Me_2_SO (+)

In order to identify roles of p38 and phospho-p38 proteins in cell differentiation, we performed Western blot analysis. Although total p38 protein was unchanged, phospho-p38 protein levels, or activated form of p38, were increased 1.8-fold on the differentiation day 4 in the presence of Me_2_SO (Fig. 5a). Importantly, phospho-p38 expression was quantitatively unchanged, and nearly stable from day 4 to day 14, which are comparable to changes of the transcription factors (Fig. 3c). On the differentiation day 14, phospho-p38 expression was highly inhibited in the presence of an inhibitor of p38 phosphorylation SB, although it was unaffected by PD and SP (Fig. 5b).

### p38-dependent expression of Csx/Nkx2.5 and GATA4

**Fig. 6:**
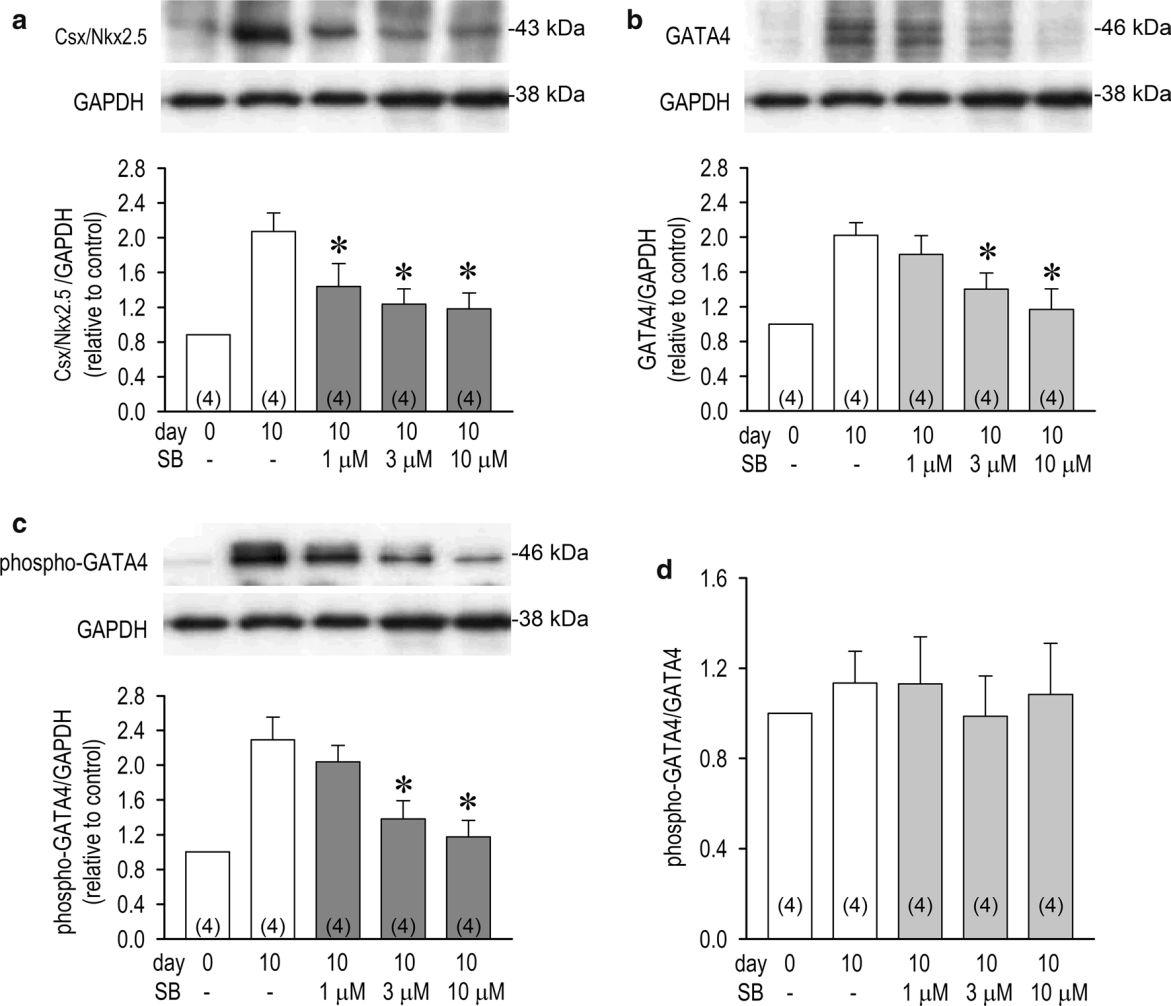
Effects of p38 MAPK inhibition on Csx/Nkx2.5 and GATA4 proteins expression. A p38 MAPK inhibitor SB203580 reduced expression of Csx/Nkx2.5 (**a**), GATA4 (**b**) and phospho-GATA4 proteins (**c**) in a concentration-dependent manner assessed by Western blot analysis in P19-derived cardiomyocytes. **d** The ratio of phospho-GATA4 proteins to total GATA4 proteins with or without SB203580 (1–10 μM) assessed by data in panels (**b**, **c**). *p < 0.01 vs. SB (−) at day 10

Unlike Csx/Nkx2.5, GATA4 is regulated to enhance its transcriptional potency by the MAP kinase signal cascade through direct phosphorylation. To further confirm and specify the role of p38 MAP kinase, protein expression of Csx/Nkx2.5 and GATA4 was examined with or without the action of p38 MAP kinase. Expression of Csx/Nkx2.5 and GATA4 proteins were significantly reduced by a p38 MAP kinase inhibitor SB in a concentration-dependent manner (Fig. 6a, b). Although expression of phospho-GATA4 was similarly inhibited by SB, decreases of phospho-GATA4 by SB were identical to those in total GATA4 as indicated by phospho-GATA4/GATA4 ratio in Fig. 6d, which suggests that p38 MAP kinase-dependent phosphorylation acts not to activate GATA4 transcriptional potency but to increase GATA4 transcription.

## Discussion

The electrical and mechanical mechanisms governing the precise and highly organized actions in the heart are extremely complex, requiring coordinated neural and humoral factors in the healthy and pathological conditions [21–31]. Such sophisticated coordination also depends on the developmental changes in the steps of cardiogenesis including the generation of pacemaker potentials [1–6, 32–34]. Our present study suggests the possible mechanism of the intracellular signal pathways that regulates expression of cardiac-specific transcription factors responsible for the generation of pacemaker potentials/ion channels in P19-derived cardiomyocytes.

### Actions of the MAP kinase pathway on differentiation of cardiomyocytes

During the cardiogenetic differentiation of P19, three distinct MAP kinases, namely, JNK, p38, and ERK1/2, are coordinately activated, and contribute to both proliferation and differentiation [15]. Our results demonstrate that P19-derived cells exert cardiomyocyte-like electrophysiological features of spontaneous beating activity with distinct expression of pacemaker ion channels under the inhibition of either ERK1/2,5 or JNK MAP kinase. These observations are consistent with a previous study demonstrating a dispensable role of “classical” MAP kinase pathway of ERK1/2 on cardiomyocyte differentiation in mouse ES cells [35]. Based on the current study, inhibition of p38 MAP kinase absolutely disrupted the cardiogenetic process of P19 cells in the following ways: (1) spontaneous beating activity was absent during the observation period of 30 days; (2) cellular membrane potentials were identical to those of undifferentiated P19 cells; and (3) current density of *I*_Ca.L_, *I*_Ca.T_ and *I*_f_ was substantially nil. Therefore, our results imply that p38 MAP kinase plays a pivotal role as the intracellular signal mediator generating pacemaker ionic channels during the process of P19-derived cardiomyocyte differentiation.

### The possible relationships between Csx/Nkx2.5 and GATA4

Several critical transcription factors, including Csx/Nkx2.5, GATA4 and MEF2C, have been shown to regulate cardiac-specific genes, with no single transcription factor responsible for the differentiation of lateral plate mesoderm into cardiac cells [8]. Recent studies have indicated the functional cooperation of Csx/Nkx2.5 and GATA4 for cardiogenetic processes [10, 14]. Based on our experiments, the interaction of Csx/Nkx2.5 with GATA4 in cardiogenesis is likely due to four possible mechanisms:

1. Csx/Nkx2.5-dependent activation of GATA4. Several studies have elucidated the critical role of Csx/Nkx2.5 during specific cardiac differentiation and have identified direct downstream targets for Csx/Nkx2.5, namely, ANP, cardiac α-actin, MHC, and others [9, 10]. The expression of Csx/Nkx2.5 was detected in cardiac crescent formation at embryonic day 7.5 (E7.5), prior to the expression of GATA4 at E8 in the formation of the linear heart tubes [8]. Under the inhibition of p38 MAP kinase, expression of Csx/Nkx2.5 was slightly maintained on day 4 or later, while expression of GATA4 was highly eliminated (Fig. 3*)*. Importantly, Csx/Nkx2.5, as a cofactor of GATA4, has been shown to be able to recruit GATA4 to cardiac gene promoters [36].
2. GATA4-dependent activation of Csx/Nkx2.5. It is notable that the extracellular signal of BMP is transformed via the interaction of Smad1/4 and GATA4 into Csx/Nkx2.5 activation [37]. Under the inhibition of p38 MAP kinase, the expression of GATA4 preceded Csx/Nkx2.5 on day 2 (Fig. 3k), indicating that GATA4 may activate the expression of Csx/Nkx2.5. Our results disclosed that on differentiation day 14, GATA4 was nearly completely inhibited while expression of Csx/Nkx2.5 persisted. It is therefore suggested that Csx/Nkx2.5 activation by GATA4 could be sustained for a short period of time.
3. Synergistic interaction of GATA4 and Csx/Nkx2.5. Several recent studies have demonstrated that both homeodomain of Csx/Nkx2.5 and DNA-binding domain of MEF2C served as cofactors that interact with the C-terminal domain of GATA4, generating a complex for synergistic transactivation of genes [11]. The most likely correct speculation is that expression of GATA4 plays a crucial role during differentiation, and that p38 MAP kinase predominantly modulates expression of GATA4, while the expression of Csx/Nkx2.5 is stimulated by dual pathways of GATA4 and other intracellular factors.
4. Independent interaction of GATA4 with Csx/Nkx2.5. The simplest explanation of our results is that GATA4 and Csx/Nkx2.5 independently regulate downstream targets and that both factors are required to activate all related cardiac ion channel genes expression needed for differentiation and cell beating. The expression of GATA4 exhibited high sensitivity to a p38 MAP kinase inhibitor, while the expression of Csx/Nkx2.5 was mediated, in a part, by the p38 MAP kinase pathway, possibly regulated by other intracellular factors such as SRF and others [11].

### MAPK unaffected MEF2C activation

The expression of MEF2C is regulated by many intracellular and intranuclear transcription factors. GATA4 may physically interact with MEF2C, thus regulating cardiac gene expression [38]. In P19CL6noggin cells, the overexpression of Csx/Nkx2.5 induced the expression of MEF2C, which suggests that expression of MEF2C was positively regulated by Csx/Nkx2.5 [10]. However, our current study could be considered partly inconsistent with these previous findings in that the expression of MEF2C was almost identical to those under control conditions utilizing the p38 MAP kinase inhibitor, whereas Csx/Nkx2.5 and GATA4 were notably abolished. During cardiogenesis, other intracellular pathways such as PI3-kinase pathway [8] could be a candidate for the MEF2C activator.

### p38 MAP kinase modulates the expression of GATA4

Several studies have disclosed that protein kinases, including ERK1/2 [39] and p38 MAP kinase [40], catalyze Ser-105-specific phosphorylation of GATA4 in cardiomyocytes. Based on our findings in Fig. 3e, ERK1/2,5 and p38 MAP kinase may be distinct in function in terms of cardiogenetic differentiation in P19 cells. Consistent with our study, in the context of rat BNP promoter, p38, but not other ERK-dependent transcriptions, is responsible for GATA4 binding site activation in the promoter region, suggesting that the activation of p38 MAP kinase is necessary for ET1-induced GATA4 activation that leads to BNP gene expression in neonatal rat cardiomyocytes [41]. Interestingly, a recent study demonstrated that p38 kinase was moderately activated by the ERK1/2,5 inhibitor PD in neonatal rat cardiomyocytes [42]. Consistent with these studies, our work demonstrates that the expression of GATA4 and Csx/Nkx2.5 was augmented under the influence of ERK1/2,5 inhibition by PD (Fig. 4), supporting our hypothesis that p38 MAP kinase-dependent activation of GATA4 and Csx/Nkx2.5 is crucial for cardiogenesis in P19 cells. This is supportive of our electrophysiological determination that *I*_Ca.L_ and *G*_max, Ca.L_ with PD were larger than those under control conditions (Fig. 2e, h). On the other hand, inhibition of the JNK pathway had no effect on ET1-induced GATA4 activation [41]. Moreover, overexpression of MEKK1, an upstream kinase of JNK, independently induced BNP promoter activation on GATA4 actions [40]. Consistently, treatment of P19 with a JNK inhibitor had no influence on the differentiation or expression of pacemaker ion channels in this study.

### Study limitation

Although we have successfully identified that activation of transcription factors Csx/Nkx2.5 and GATA4 is a trigger for pacemaker ion channels expression including Cav3.2-T-type Ca^2+^ channel regulation [43], we need further investigation to clarify these possible association with other transcription factors such as Tbx3 and Tbx18, which reportedly form sino-atrial node in the early cardiogenesis [44]. Although the generation of cardiac pacemaker potentials relies on a complex interplay between different types of currents carried by ion channels/transporters, we only focused on the three major ion channels on this study. Moreover, we were unable to identify molecules responsible for the automaticity in P19-derived cardiomyocytes. In cardiac pacemaker cells, the spontaneous action potential and resulting contraction is brought about through time- and voltage-dependent changes in the gating of ionic channels. The alternative activation of inward and outward current systems produces a feedback loop which generates the oscillation of the membrane potential. A large number of ionic currents in single sinus node cells have been identified, and, using the conductance and gating parameters of the individual current components, it is possible to reproduce the pacemaker activity by computer simulation. For instance, an example of the pacemaker model simulation, in which the action potential and ionic currents in sinus node cells were reproduced using the Kyoto Model developed by Sarai et al. [45]. According to their study, more than 13 ionic currents have been incorporated. The currents include L-(I_CaL_) and T-type Ca^2+^ currents (I_CaT_), the delayed rectifier K^+^ current (I_K_), Na^+^-K^+^ pump current (I_NaK_), muscarinic K^+^ current (I_K,ACh_), hyperpolarization-activated cation current (If), background cation current and a dihydropyridine-sensitive sustained inward current (I_st_), and others. It is generally accepted that the rising phase of the action potential is due to I_CaL_ and that the repolarization is caused by the inactivation of I_CaL_ and the activation of I_K_. As the membrane hyperpolarizes, I_K_ begins to deactivate, which in turn triggers a gradual depolarization after the maximum diastolic potential. During the later part of the pacemaker potential, several inward currents such as If, I_CaT_ and I_st_ participate in driving the membrane towards the threshold of the following action potential. Among them, Cav1.3-L-type Ca^2+^ channel and HCN2/4-If channel could be major contributors [46–48], although we were unable to confirm their molecules in this P19-derived cardiomyocyte. These are the description on the classical “membrane clock”. Recent experimental evidence based on confocal cell imaging and supported by numerical modeling, however, shows that initiation of cardiac impulse is a more complex phenomenon and involves yet another clock that resides under the sarcolemma [49]. This clock is the sarcoplasmic reticulum (SR): it generates spontaneous, but precisely timed, rhythmic, submembrane, local Ca^2+^ releases that appear not at the maximum diastolic potentials but during the late diastolic depolarization. We could not confirm molecules for the Ca^2+^ clock either in this investigation. Nevertheless, transcriptional regulation of other ion channels/transporters such as K^+^ channels, Na^+^ channels, Cl^−^ channels and Na^+^–Ca^2+^ exchanger by Csx/Nkx2.5 and GATA4 needs to be elucidated. In addition, pacemaker rhythm in this cardiogenetic model is much slower than the beating ratio of mouse sino-atrial node, which requires further investigations to identify molecules needed to tune and mature pacemaking cardiomyocytes.

## Conclusions

Our results demonstrate that the transcription factors Csx/Nkx2.5 and GATA4 association with p38 MAP kinase but no other MAP kinases regulates transcription of pacemaker ion channels contributing to spontaneous beating activity during the process of cardiac myocyte differentiation.

## Data Availability

The datasets generated during and/or analyzed during the current study are available in the Oita University School of Medicine repository. They are also available from the corresponding author on reasonable request.

## Abbreviations

MAP: Mitogen-activated protein
I_f_: Hyperpolarization-activated inward current
I_Ca.L_: L-type Ca^2+^ channel current
I_Ca.T_: T-type Ca^2+^ current
ERK: Extracellular signal-regulated kinase
I_Kr_: Rapidly activating delayed rectifier K^+^ current
I_st_: Sustained inward current
Me_2_SO: Dimethyl sulfoxide
I_to_: Transient outward K^+^ current
bpm: Beat per minute
BR: Beating rates
MDP: Maximum diastolic potentials
APD_50_: The duration of 50% repolarization of action potentials
APD_75_: The duration of 75% repolarization of action potentials
V_1/2_: Half-activated potentials
C_m_: Cellular membrane capacitance
G_max_: The maximum chord conductance
